# High-Stability Silver Nanowire–Al_2_O_3_ Composite Flexible Transparent Electrodes Prepared by Electrodeposition

**DOI:** 10.3390/nano11113047

**Published:** 2021-11-12

**Authors:** Honglong Ning, Junlong Chen, Zhihang Li, Zhuohui Xu, Rihui Yao, Hongfu Liang, Taijiang Liu, Guoping Su, Dongxiang Luo, Junbiao Peng

**Affiliations:** 1 State Key Laboratory of Luminescent Materials and Devices, Institute of Polymer Optoelectronic Materials and Devices, South China University of Technology, Guangzhou 510640, China; ninghl@scut.edu.cn (H.N.); msjlchen@gmail.com (J.C.); mslzhscut@mail.scut.edu.cn (Z.L.); 201530291429@mail.scut.edu.cn (H.L.); ltjscut@163.com (T.L.); 201730321254@mail.scut.edu.cn (G.S.); psjbpeng@scut.edu.cn (J.P.); 2 Guangxi Key Lab of Agricultural Resources Chemistry and Biotechnology, Yulin Normal University, Yulin 537000, China; xzh21@ylu.edu.cn; 3 Laboratory for Clean Energy and Materials, Guangzhou Key Huangpu Hydrogen Innovation Center, School of Chemistry and Chemical Engineering, Institute of Clean Energy and Materials, Guangzhou University, Guangzhou 510006, China

**Keywords:** silver nanowires, Al_2_O_3_, electrodeposition, flexible transparent electrodes, high-stability

## Abstract

Silver nanowire (AgNW) conductive film fabricated by solution processing was investigated as an alternative to indium tin oxide (ITO) in flexible transparent electrodes. In this paper, we studied a facile and effective method by electrodepositing Al_2_O_3_ on the surface of AgNWs. As a result, flexible transparent electrodes with improved stability could be obtained by electrodepositing Al_2_O_3_. It was found that, as the annealing temperature rises, the Al_2_O_3_ coating layer can be transformed from Al_2_O_3_·H_2_O into a denser amorphous state at 150 °C. By studying the increase of electrodeposition temperature, it was observed that the transmittance of the AgNW–Al_2_O_3_ composite films first rose to the maximum at 70 °C and then decreased. With the increase of the electrodeposition time, the figure of merit (FoM) of the composite films increased and reached the maximum when the time was 40 s. Through optimizing the experimental parameters, a high-stability AgNW flexible transparent electrode using polyimide (PI) as a substrate was prepared without sacrificing optical and electrical performance by electrodepositing at −1.1 V and 70 °C for 40 s with 0.1 mol/L Al(NO_3_)_3_ as the electrolyte, which can withstand a high temperature of 250 °C or 250,000 bending cycles with a bending radius of 4 mm.

## 1. Introduction

With the development of flexible devices, indium tin oxide (ITO) cannot always be used as flexible transparent electrode due to its fragility and its high preparation temperature that may damage flexible substrates. New types of transparent conductive films suitable for flexible substrates such as silver nanowires (AgNWs) [1], carbon nanotubes [2], graphene [3], and metal grids [4] have begun to emerge. Among them, the AgNW conductive film fabricated by solution processing can not only achieve better optical and electrical performance than ITO, but can also be compatible with the low-temperature preparation process required for flexible display [5,6,7]. AgNWs have been used in optoelectronic devices such as organic light-emitting diode (OLED) [8,9], touch screen [10], solar cell [11], and electrochromic devices [12,13]. However, AgNWs still have to solve several problems such as reliability, which are mainly manifested in the following aspects. (1) Thermal stability: At the nanoscale, the melting point of the material will drop sharply [14]. The radius of the nanowires and external environmental factors such as temperature play an important role in the stability of AgNWs [15]. (2) Electrical stability: Unlike ITO that is transmitted through a surface, electrons only move through a cross-section with a diameter of about 50 nm in the AgNWs. This will generate a large amount of Joule heat and make the conductive network ineffective [16]. (3) Contact reliability: It is difficult for AgNWs coated by the solution method to form a strong bond with the substrate, resulting in the problems of easy shedding and heat transfer. Heat treatment is an effective method to improve contact [17]. However, reaching the temperature to improve the contact may cause the thermal failure of the AgNWs network.

To improve the stability of the AgNWs, the common method is to form a protective layer on the surface of the AgNWs by the sol-gel method, vacuum deposition method, coating with oxide nanoparticles or organics, and so on. However, such reliability improvement schemes cannot avoid performance degradation or cost increase. The electrodeposition method has the characteristics of low cost and a simple process, showing the potential to solve this problem. Moreover, this method has almost no effect on the open area through which light passes, which can be considered as an ideal method for the surface protection of AgNWs [18,19]. Lee [20] and Kang [21] et al. directly electrodeposited silver on the AgNW network to strengthen the contact at the node. However, this method increases the light absorption and surface roughness of the AgNW network at the same time. Yang et al. [22] formed a protective layer by electrodepositing graphene and then laser processing, which showed good high-temperature resistance and high-voltage resistance. Zhang et al. [23] used magnetic field assistance to electrodeposit nickel on the AgNWs, which effectively improved the adhesion of the conductive film. However, the methods in existing reports basically lead to the increase in the surface roughness of AgNWs, resulting in a decrease in transmittance. Furthermore, the introduction of graphene laser processing and magnetron electrodeposition increases the cost and difficulty of operation, so it is urgent to find a new electrodeposition process that takes into account both cost and efficiency.

In this paper, we propose a facile and effective method by electrodepositing Al_2_O_3_ on the surface of AgNWs, which can significantly improve the electrical, thermal, and mechanical stability of the flexible transparent electrode.

## 2. Materials and Methods

### 2.1. Synthesis of Silver Nanowires

The AgNWs were synthesized with a modified polyol method. Polyvinylpyrrolidone (PVP, M_w_ ≈ 360,000, Shanghai Macklin Biochemical Co., Ltd., Shanghai, China) was added to ethylene glycol (EG, Shanghai Richjoint Co., Ltd., Shanghai, China) and heated to 160 °C with vigorous stirring. After 30 min, 26.88 mmol/L of NaCl (Shanghai Richjoint Co., Ltd., Shanghai, China) and 9 mmol/L of NaBr dissolved in EG were added to the mixture and stirred for another 30 min. Then, 137.95 mmol/L of AgNO_3_ (Sinopharm Chemical Reagent Co., Ltd., Shanghai, China) dissolved in EG was added dropwise to the mixture for 10 min. Next, the mixture was kept at 160 °C for 1 h without stirring until the reaction finished. After cooling, the solution was purified with acetone and ethanol, and suspended in ethanol. AgNWs dispersion in ethanol was spin-coated on a substrate then annealed at 110 °C for 30 min to fabricate transparent electrodes for electrodeposition (samples were flat on a hot stage at air atmosphere with standard atmospheric pressure, all other thermal annealing methods described in this paper are the same except for temperature and time).

### 2.2. Electrodeposition of Al_2_O_3_

For electrodeposition, 0.1 mol/L of Al(NO_3_)_3_ (Tianjin Kermel Co., Ltd., Tianjin, China) was dissolved in deionized water. The above-mentioned AgNW transparent electrode, 20 mm × 20 mm Pt sheet, and Ag|AgCl electrode were used as the working electrode, the counter electrode, and the reference electrode, respectively. A constant potential of −1.1 V with respect to the reference electrode was applied up to a certain time using a potentiostat (Corrtest CS1002 constant potential/constant current meter). After electrodeposition, the electrodeposited film was rinsed by immersing it in stirred deionized water to remove impurities. Then, the sample was dried on a hot stage at 80 °C for 15 min to complete the preparation of the electrodeposited AgNW–Al_2_O_3_ transparent electrode. The following Reactions (1) and (2) occur during the electrodeposition process [24]:

NO_3_^−^ + H_2_O + 2e^−^→NO_2_^−^ + 2OH (1)

2Al^3+^ +6OH^−^→Al_2_O_3_ + 3H_2_O (2)

### 2.3. Characterization

The scanning electron microscope (SEM) images were taken by a Hitachi Regulus8100 field-emission SEM, and the morphology was observed with an Olympus 3D confocal laser scanning microscope (CLSM) OLS5000 (Olympus Corporation, Tokyo, Japan). The flexible performance of the transparent electrode was measured using a CRYSCO bending life tester (Guangzhou Jinghe Co., Ltd., Guangzhou, China). The sheet resistance was tested by a Guangzhou Four-Probe Technology RTS-9 double-electric four-probe tester. The electrical stability was measured using a National Instruments PXIe-1071 dual-channel high-precision electrical performance tester and microprobe station. The transmittance of the sample and the transmission spectrum were achieved using a Shimadzu UV-2600 ultraviolet-visible spectrophotometer. The composition and crystal structure of the sample were analyzed using a PANalytical Empyrean X-ray diffractometer (XRD). A Nexsa X-ray Photoelectron Spectrometer System (Thermo Fisher Scientific, Waltham, USA) was used for X-ray photoelectron spectrometry (XPS).

## 3. Results and Discussion

**Figure 1:**
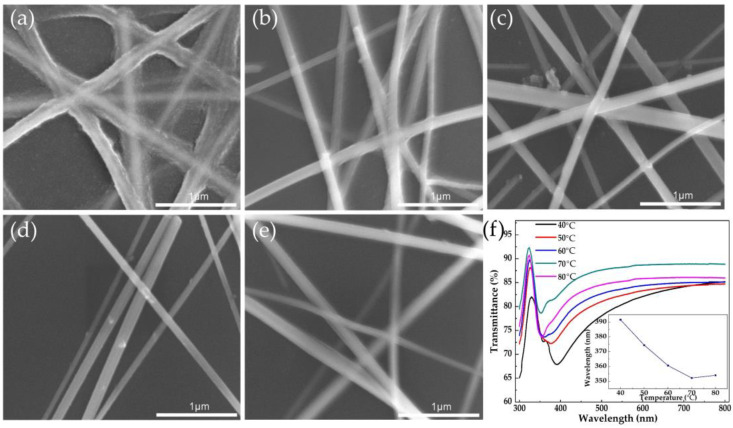
SEM images of AgNWs’ electrodeposited Al_2_O_3_ at different temperatures for 40 s: (**a**) 40 °C, (**b**) 50 °C, (**c**) 60 °C, (**d**) 70 °C, and (**e**) 80 °C. (**f**) UV-visible transmittance spectra of products at different temperatures; inset shows the change of absorption peak with temperature.

To analyze the influence of Al_2_O_3_ coating on the optical properties of AgNWs at different deposition temperatures, the electrodeposition time was fixed at 40 s and 5 min and the samples were reacted at 40 °C, 50 °C, 60 °C, 70 °C, and 80 °C, respectively. The morphology of the product is shown in Figure 1 and Figure S1. At 40 °C, AgNWs were wrapped by an electrodeposited layer with a thickness equivalent to one wire diameter after only 40 s, and they were completely covered by the entire film when it was deposited for 5 min. At 50 °C, uneven protrusions could be observed on the surface of the AgNWs, and a discontinuous film covered the area without AgNWs at 5 min. When the temperature rose to 60 °C, there were a few particles attached to the surface of the AgNWs at 40 s and the surface of AgNWs and substrate were covered with a deposited layer of hundreds of nanometers at 5 min. At 70 °C, only a small number of particles could be observed on the surface. Even if the reaction was increased to 5 min, only a slight increase in the surface roughness of the AgNWs appeared, and there was almost no effect on the area without AgNWs. The situation at 80 °C was similar to that at 70 °C. It can be found from the transmission spectra of Figure 1f that the transmittance of the film obtained by electrodeposition at 70 °C was the highest, and the absorption peak at 70 °C during the deposition for 40 s had the shortest wavelength. The absorption peak is usually determined by the local surface plasmon resonance of the nanowires, reflecting the structure of the smaller wire diameter nanowires [25]. The blue shift of the absorption peak is beneficial to reduce the absorption of visible light to meet the needs of high-performance flexible transparent electrodes.

**Figure 2:**
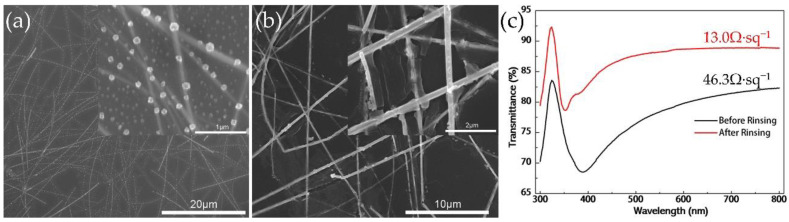
SEM images of AgNW–Al_2_O_3_ composite film (electrodeposition time and temperature: 40 s @ 70 °C) (**a**) before rinsing and (**b**) after rinsing. (**c**) UV-visible transmission spectra before and after rinsing.

The SEM images and ultraviolet (UV)-visible transmission spectra of AgNW–Al_2_O_3_ composite film are shown in Figure 2. From Figure 2a, the unrinsed electrodeposited Al_2_O_3_ film residue formed a large number of particles, which became light scattering centers and affected the transmittance of the film (78.4% before rinsing and 85.31% after rinsing). Besides, the sheet resistance of the film greatly increased due to contact problems (46.3 Ω/sq before rinsing and 13.0 Ω/sq after rinsing). To remove impurities, the electrodeposited film was immersed in stirred deionized water and rinsed for 30 s. After rinsing, the electrodeposited layer on the surface was a relatively smooth continuous film. From Figure 2b, a coating layer with holes can be observed on the surface of the AgNWs, and there was a sheet-like film on the surface of the layer. According to the Frenkel–Poole effect [26], the insulating layer below a certain size is prone to form interface defect states and forms a defect band in the forbidden band for electrons to flow, which makes Al_2_O_3_ conductive [27].

To analyze the effect of the Al_2_O_3_ cladding layer on improving the electrothermal stability of AgNWs under different deposition times, the reaction temperature was maintained at 70 °C and the composite films electrodeposited for 0 s, 10 s, 20 s, and 40 s were continuously heated to 150 °C, 200 °C, 250 °C, and 300 °C for 30 min each, to evaluate the protective effect of Al_2_O_3_ by the change of sheet resistance. The optical and electrical properties of the film before and after electrodeposition are shown in Table 1. The following equation was used to fit the figure of merit (FoM) [28]:

FoM = T^10^/R_s_·1000 (3)

where T is the transmittance at 550 nm and R_s_ is the sheet resistance. The higher the FoM, the better the performance of the transparent electrode. The electrodeposition of Al_2_O_3_ not only did not reduce the performance as a transparent conductive film but even improved the figure of merit of the composite film [29]. Since Al_2_O_3_ at the nanoscale is an almost transparent material under visible light and only covers the surface of the silver nanowires, it has little effect on the optical transmittance of the film. In the process of deposition and drying, the capillary force generated by the Al_2_O_3_ coating and high surface tension water during the drying process strengthens the contact between the AgNWs and reduces the node resistance.

**Figure 3:**
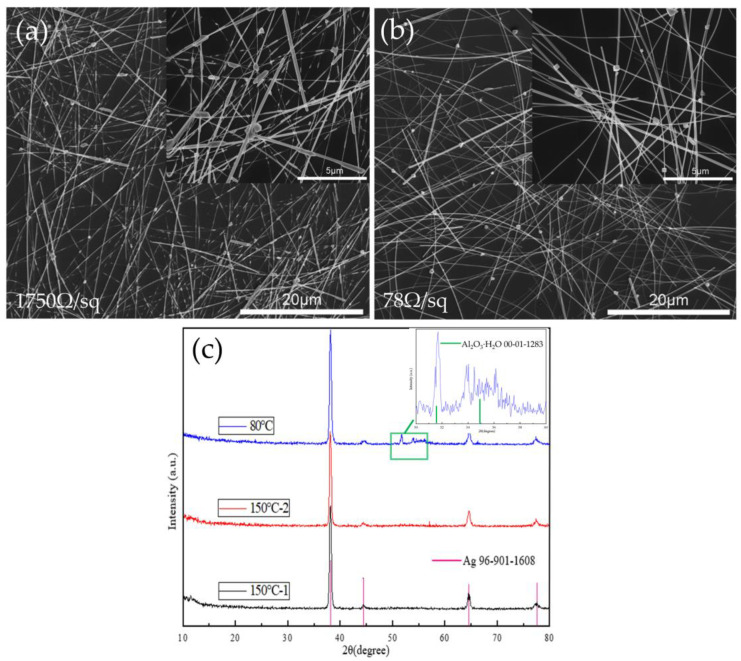
SEM images of AgNW–Al_2_O_3_ composite films with different deposition times after heat treatment at 200 °C for 30 min: (**a**) 20 s and (**b**) 40 s. (**c**) XRD pattern of the composite film before and after annealing at 150 °C for 30 min; inset shows the enlarged pattern in the box.

The AgNW–Al_2_O_3_ composite film was directly heated on a 200 °C hot stage for 30 min, which is different from the stepped heating mentioned above. The sheet resistance of the composite films increased sharply by about two orders of magnitude for the films electrodeposited for 20 s (from 18 Ω/sq to 1750 Ω/sq) and by a factor of three for the films electrodeposited for 40 s (from 19.7 Ω/sq to 78 Ω/sq). The SEM images in Figure 3 show that the film electrodeposited for 20 s had begun to spheroidize, most of the nanowires became thinner and broke, and Ag tended to accumulate at the nodes. The films electrodeposited for 40 s recrystallized by forming tetrahedral and hexahedral particles, with a local fracture of AgNWs. The difference between the two treatment methods was whether there was a 150 °C treatment for 30 min. The surface energy of Al_2_O_3_ crystals is 1.5~2.64 J/m^2^ [30], while the surface energy of amorphous Al_2_O_3_ is 0.97 ± 0.04 J/m^2^ [31], which has a higher stability. Through the XRD characterization of Figure 3c, it can be found that Al_2_O_3_·H_2_O (AlOOH) was present before the treatment at 150 °C. The coating layer in this state cannot effectively protect the AgNWs. However, heat treatment at 150 °C can transform it into a denser amorphous state, suppressing the thermal failure of the AgNWs.

**Figure 4:**
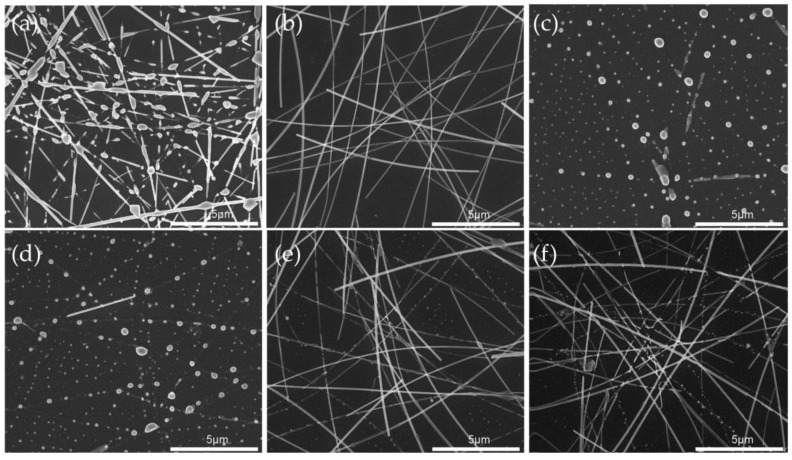
SEM images of films with different deposition time after heat treatment at different temperature: (**a**) pure AgNWs at 200 °C and AgNW–Al_2_O_3_ composite films at (**b**) 200 °C@40 s, (**c**) 300 °C@10 s, (**d**) 300 °C@20 s, (**e**) 300 °C@30 s, and (**f**) 300 °C@40 s.

**Figure 5:**
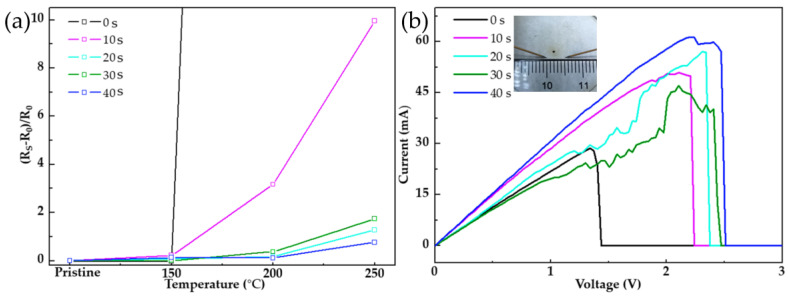
Thermal and electrical stability of films with different deposition times: (**a**) relationship between sheet resistance and temperature and (**b**) the change of failure voltage.

Without reducing the optical and electrical performance, the AgNW–Al_2_O_3_ composite film prepared in this study also showed higher stability. As shown in Figure 4, after heat treatment at 150 °C and 200 °C for 30 min each, due to the hot spot effect at the nodes, the AgNWs without am electrodeposited Al_2_O_3_ coating layer were spheroidized and disconnected under the action of Rayleigh instability. At this time, the film lost its electrical conductivity. However, the AgNW–Al_2_O_3_ composite films electrodeposited for 40 s after the same heat treatment showed no significant difference under SEM, and the sheet resistance only increased by 11.5%. As shown in Figure 5a, the AgNWs electrodeposited by Al_2_O_3_ can withstand a temperature rise above 250 °C, and the effect of this stability improvement tended to ease with the increase of the reaction time. When heated to 300 °C, while the AgNWs in the films electrodeposited for 10 s and 20 s had undergone complete spherization, most of the AgNWs in the films for 30 s and 40 s still retained their original structure, indicating that the Al_2_O_3_ electrodeposited layer can effectively avoid the thermal failure of the AgNWs. Similar to the electrical failure caused by Joule heat accumulation at the node, it can also be suppressed by the Al_2_O_3_ electrodeposition layer. By testing the I-V curves at two points 5 mm apart on the film, the composite film improved the failure voltage by more than 60% compared to the AgNWs without electrodeposition (Figure 5b).

**Figure 6:**
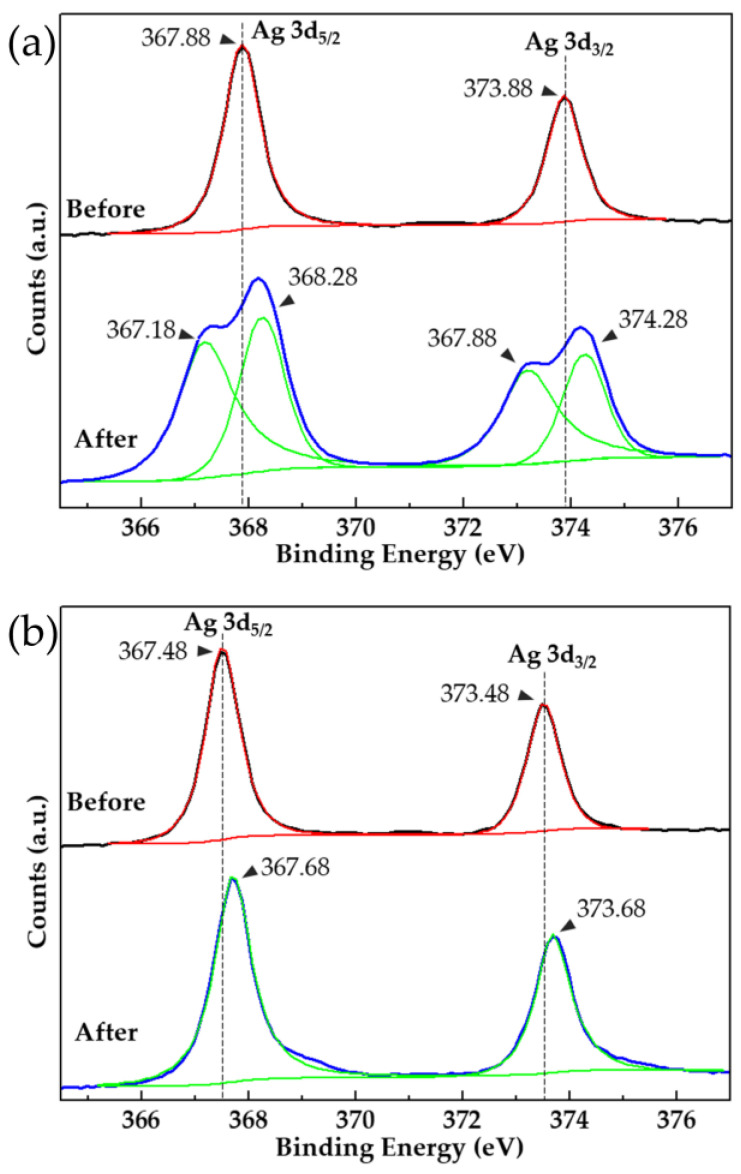
Ag3d XPS spectra before and after heat treatment at 250 °C for 30 min: (**a**) pure AgNWs and (**b**) AgNW–Al_2_O_3_ composite film.

As shown in the XPS spectrum of Figure 6, after the AgNWs were heat-treated at 250 °C for 30 min, the doublet in the Ag3d spectrum split into four peaks. Among them, the two peaks of energy decrease were caused by the formation of Ag_2_O [18]. The AgNWs after 40 s of electrodeposition did not undergo the above changes after heat treatment, and the binding energy only slightly increased by 0.2 eV. In addition, before the heat treatment, the binding energy of the electrodeposited AgNWs decreased by 0.4 eV, the outer electron cloud density increased, and the Ag transitioned to a low valence state, indicating that the oxidation of the AgNWs in the air atmosphere was also inhibited. For one thing, the core–shell structure wrapped by the more stable oxide limits the spheroidization deformation of the AgNWs at high temperatures. For another, the electrodeposited Al_2_O_3_ between the AgNWs and the filling between the nanowires and the substrate provide a conductive channel for the AgNWs, which avoids the accumulation of heat at the nodes and improves the heat resistance of the conductive network of the AgNWs.

**Figure 7:**
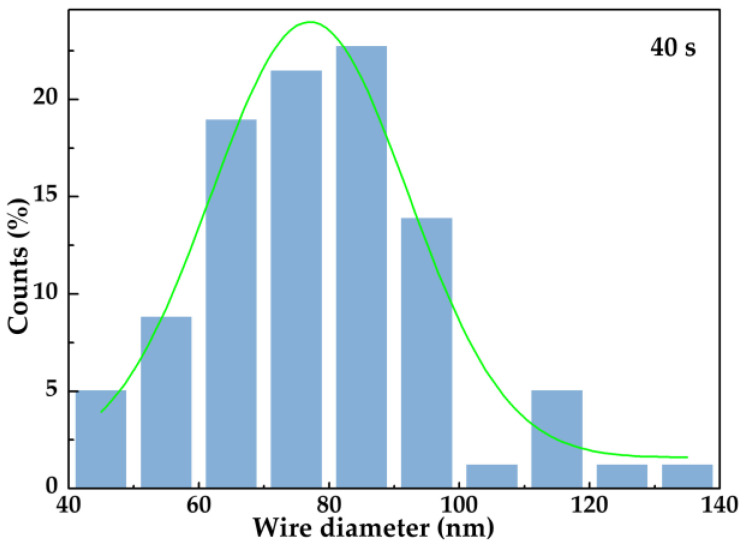
Wire diameter distribution of AgNWs deposited for 40 s.

The wire diameter distribution of AgNWs electrodeposited for 40 s in the SEM image is shown in Figure 7. The median diameter of AgNWs with a coating layer was 78.8 nm compared to that at 67.9 nm of pure AgNWs. It can be inferred that the thickness of the Al_2_O_3_ layer was about 5 nm when it was deposited for 40 s. To improve the response strength of the test, the electrodeposition time was increased to 90 s. As shown in the Al2p XPS spectrum of Figure S2, the peak value was 74.55 eV, which is consistent with the binding energy of Al_2_O_3_.

**Figure 8:**
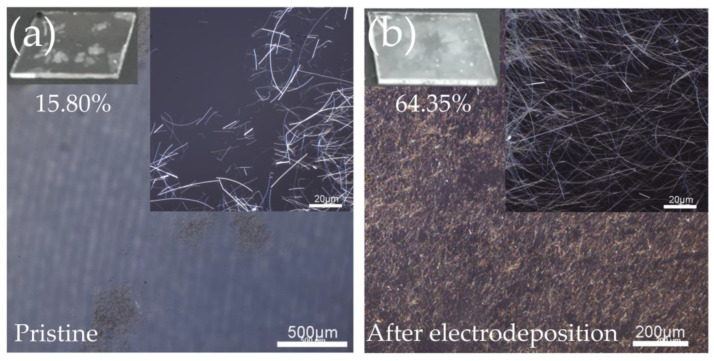
CLSM images of the films after ultrasonic treatment for 5 min: (**a**) pure AgNWs and (**b**) AgNW–Al_2_O_3_ composite film.

Through research, we found that the electrodeposited Al_2_O_3_ coating layer can improve the adhesion of AgNWs to the substrate. To test the effect of electrodeposition on enhancing the adhesion of AgNWs, the AgNWs without electrodeposition and electrodeposited for 40 s were immersed in deionized water and treated in an ultrasonic cleaner for 5 min. The results are shown in Figure 8 and Table S1, where the retention ratio (R) is calculated by the transmittance, that is,

R = (1 − T)/(1 − T_0_) (4)

Due to the different impact forces received at different positions of the AgNWs under ultrasound, they will bend and break [32]. After 5 min of ultrasonic treatment, only about 16% of the AgNWs without electrodeposition remained. The AgNWs were peeled off in sheets and no longer had the ability to conduct electricity. Although the quality factor of the AgNWs after electrodeposition of Al_2_O_3_ for 40 s dropped by about 80%, it still had a retention ratio of 60~70%. The Al_2_O_3_ significantly enhanced the adhesion of the AgNWs and retained good electrical conductivity despite the crack-like peeling in some areas.

**Figure 9:**
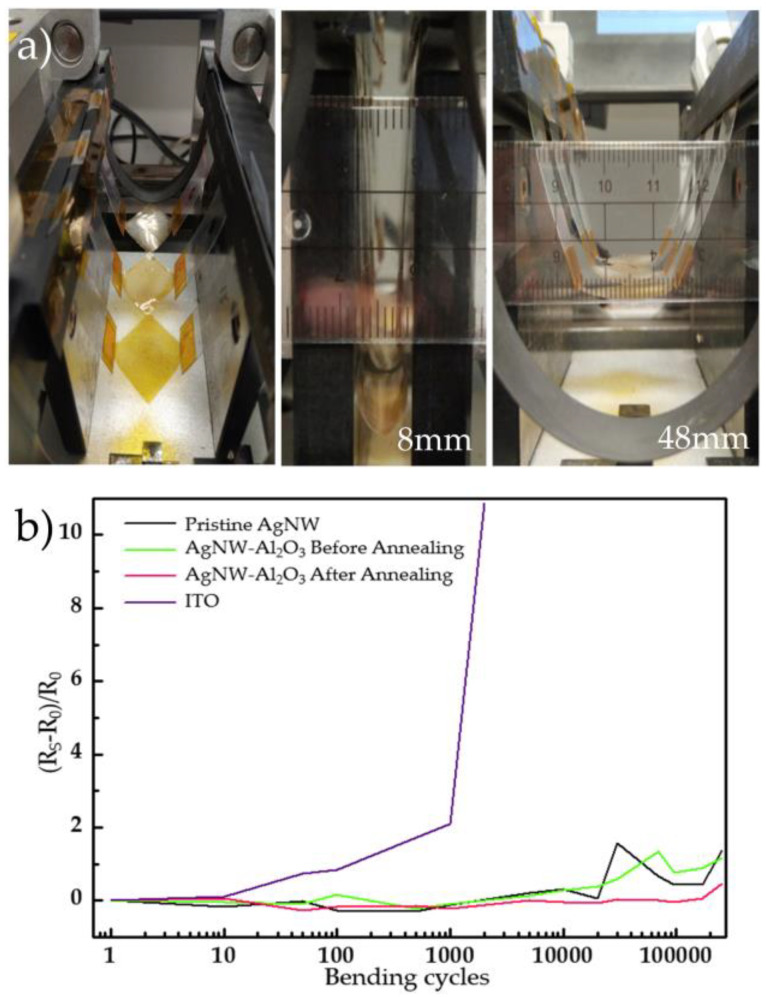
(**a**) Flexible bending test with a bending diameter of 8 mm, offset of 40 mm and (**b**) sheet resistance versus bending cycles in 250,000 bending cycles.

AgNW–Al_2_O_3_ composite films were prepared by electrodeposition at 70 °C for 40 s on a 20 mm × 20 mm polyimide (PI) substrate. The flexibility performance of AgNWs without electrodeposition, electrodeposition without annealing, 150 °C annealed electrodeposited films, and polyethylene terephthalate (PET) films deposited with ITO were tested with a bending diameter of 8 mm, offset of 40 mm, inward bending of the films, a moving speed of 150 mm/s, and a delay time of 0.1 s. The sheet resistance of ITO reached nearly 11 times that of the original one after 2000 bends, with the surface covered with transverse cracks perpendicular to the bending direction as shown in Figure S3c, and the local mesh-like cracks as shown in Figure S3a. Figure S3b shows a 3-dimensional scan of the ITO surface, and the cracks showed a 10–30 nm high bump. As for the AgNWs, as shown in Figure S3d, the annealed electrodeposited nanowires maintained the original conductive network structure after 250,000 bending cycles, and their properties were more stable. As shown in Figure 9b and Table S2, the sheet resistance increased only 5% after 170,000 bending cycles. After 250,000 bending cycles, the sheet resistance of the electrodeposited 150 °C annealed films increased by 47%, while the electrodeposited unannealed film and AgNWs without electrodeposition improved by 117% and 138%, respectively. Therefore, the annealed AgNW–Al_2_O_3_ composite films can meet the current requirements of transparent electrodes for flexible electronics.

**Figure 10:**
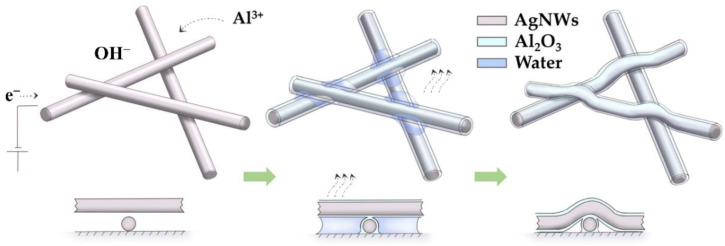
Schematic of the process of Al_2_O_3_ electrodeposition on AgNWs.

In summary, the protection principle of AgNWs’ electrodeposition with Al_2_O_3_ can be described by the schematic diagram in Figure 10. The improvement in adhesion and flexibility can be attributed to the electrodeposition of Al_2_O_3_ between the AgNWs. The filling between the nanowires and the substrate enhances the contact of the AgNWs, and at the same time reduces the damage to the AgNWs caused by the heat generated by bending. During the heat treatment, the evaporation of the residual aqueous solution will generate capillary forces at the AgNWs interstices [33], further promoting the contact between the AgNWs and between the AgNWs and the substrate. Meanwhile, a coalescent layer is formed that serves as joints between the otherwise separated wires, increasing the electrical and thermal conduction pathways. In addition, the presence of the Al_2_O_3_ protective layer enables the AgNWs to withstand higher annealing temperatures and promotes the welding of AgNW nodes, resulting in tighter connections between AgNWs with better resistance to bending. Moreover, defects or non-uniformities occurring during electrodeposition in conducting nanowire networks can substantially impact the electrical, optical, and mechanical properties of devices [34,35]. 

## 4. Conclusions

In this study, we proposed an electrodeposition method to prepare high-stability AgNW flexible transparent electrodes. The influence of process parameters such as rinsing, annealing, reaction temperature, and reaction time on the electrodeposited Al_2_O_3_ on the surface of the AgNWs was studied. The results show that by using 0.1 mol/L Al(NO_3_)_3_ as the electrolyte, electrodepositing at −1.1 V and 70 °C for 40 s, and annealing at 150 °C for 30 min, high-stability AgNW flexible transparent electrodes were prepared, which can significantly improve the electrical, thermal, and mechanical stability of AgNWs: (1) for the electrical stability, the failure voltage of the electrodes was increased by more than 50%; (2) for the thermal stability, the electrodes could withstand a high temperature of 250 °C without spheroidization failure; and (3) for mechanical stability, the electrodes could withstand 250,000 bending cycles with a bending radius of 4 mm at room temperature. At the same time, due to the selective deposition of the electrodeposition method and the high-flatness Al_2_O_3_ coating layer, the AgNW–Al_2_O_3_ composite film has the optical and electrical performance equivalent to that of the AgNWs, while removing the problems of the protective layer weakening the performance of AgNWs with a convenient and low-cost solution.

## Figures and Tables

**Table 1:** Optical and electrical properties at different deposition time.

Time/s	Sheet Resistance/Ω·sq^−1^	Transmittance/%	FoM/Ω^−^^1^
0	25.0	89.15	12.68
10	25.7	89.67	13.08
20	19.3	87.72	13.98
40	20.0	89.58	16.64

## Supplementary Materials

The following are available online at https://www.mdpi.com/article/10.3390/nano11113047/s1, Figure S1: SEM images of electrodeposited Al_2_O_3_ at different temperatures for 5 min: (a) 40 °C; (b) 50 °C; (c) 60 °C; (d) 70 °C; and (e) 80 °C and (f) UV-visible transmittance spectra of products at different temperatures, Figure S2: Al2p XPS spectra of films deposited for 40 s and 90 s, Figure S3: CLSM image of films after bending test: (a) reticulated cracks; (b) 3D scanning; (c) transverse cracks of the ITO-PET film after 2000 bending cycles; and (d) 3D scanning image of AgNWs–Al_2_O_3_ composite film after 250,000 bending cycles, Table S1: Changes in the optical and electrical properties of the films before and after ultrasonic treatment for 5 min, Table S2: Relationship between sheet resistance change rate and bending cycles.
