# Supplementary material for: High-Stability Silver Nanowire–Al2O3 Composite Flexible Transparent Electrodes Prepared by Electrodeposition
Source: Nanomaterials (Basel). 2021 Nov 12;11(11):3047. doi: 10.3390/nano11113047 (PMC8621956; doi:10.3390/nano11113047)
Supplement: Supplementary file 1 [file nanomaterials-11-03047-s001.zip › nanomaterials-1424208-supplementary.pdf]

nanomaterials

Supporting Material

High-Stability Silver Nanowire–Al_2O_3 Composite Flexible Transparent Electrodes Prepared by Electrodeposition

Honglong Ning [1], Junlong Chen [1], Zhihang Li [1], Zhuohui Xu [2], Rihui Yao [1,*], Hongfu Liang [1], Taijiang Liu [1], Guoping Su [1], Dongxiang Luo [3,*] and Junbiao Peng [1]

1 State Key Laboratory of Luminescent Materials and Devices, Institute of Polymer Optoelectronic Materials and Devices, South China University of Technology, Guangzhou 510640, China; ninghl@scut.edu.cn (H.N.); msjlchen@gmail.com (J.C.); mslzhscut@mail.scut.edu.cn (Z.L.); 201530291429@mail.scut.edu.cn (H.L.); ltjscut@163.com (T.L.); 201730321254@mail.scut.edu.cn (G.S.); psjbpeng@scut.edu.cn (J.P.)
2 Guangxi Key Lab of Agricultural Resources Chemistry and Biotechnology, Yulin Normal University, Yulin 537000, China; xzh21@ylu.edu.cn
3 Laboratory for Clean Energy and Materials, Guangzhou Key Huangpu Hydrogen Innovation Center, School of Chemistry and Chemical Engineering, Institute of Clean Energy and Materials, Guangzhou University, Guangzhou 510006, China
* Correspondence: yaorihui@scut.edu.cn (R.Y.); luodx@gdut.edu.cn (D.L.)

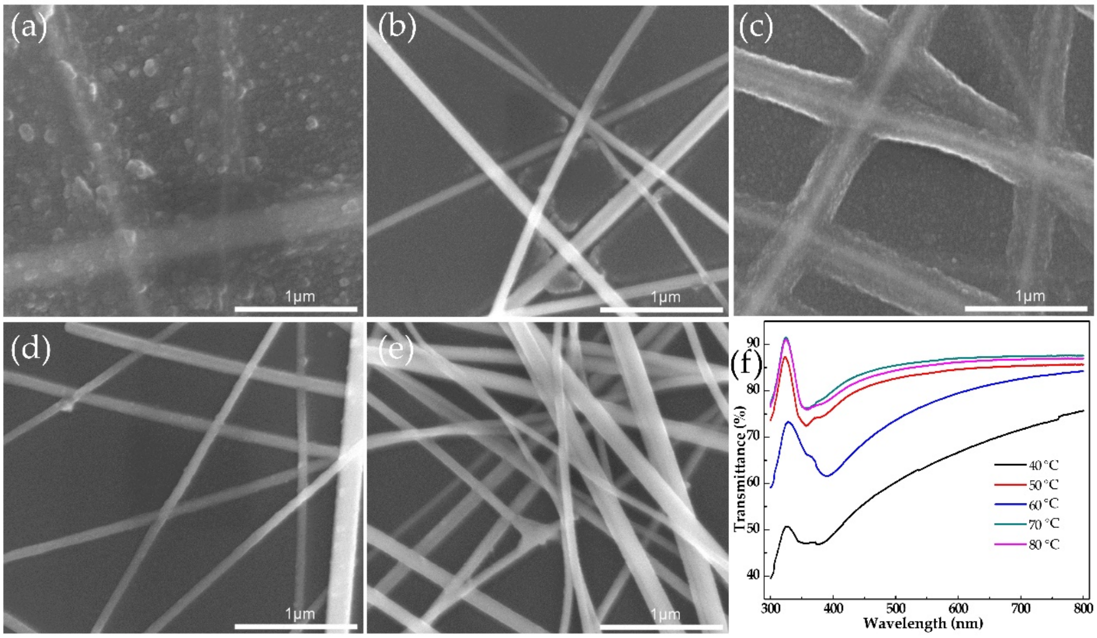

Figure S1. SEM images of electrodeposited Al_2O_3 at different temperatures for 5 min: (**a**) 40 °C; (**b**) 50 °C; (**c**) 60 °C; (**d**) 70 °C; (**e**) 80 °C and (**f**) UV-Visible transmittance spectra of products at different temperatures.

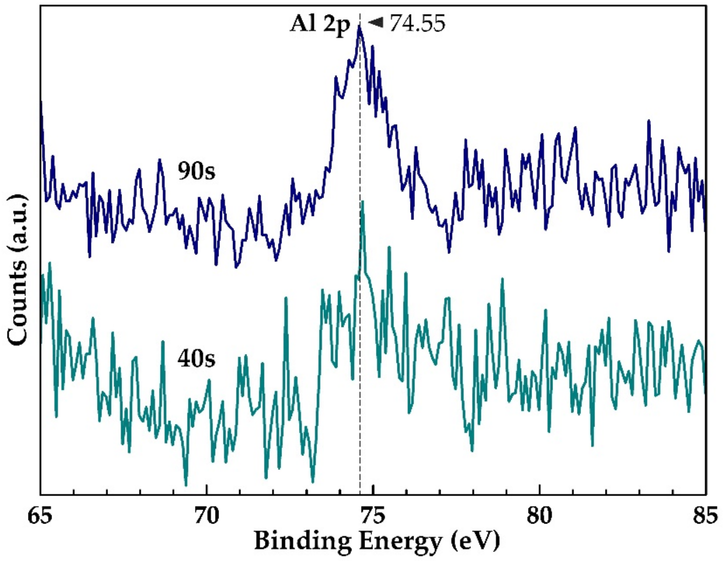

Figure S2. Al2p XPS spectra of films deposited for 40 s and 90 s.

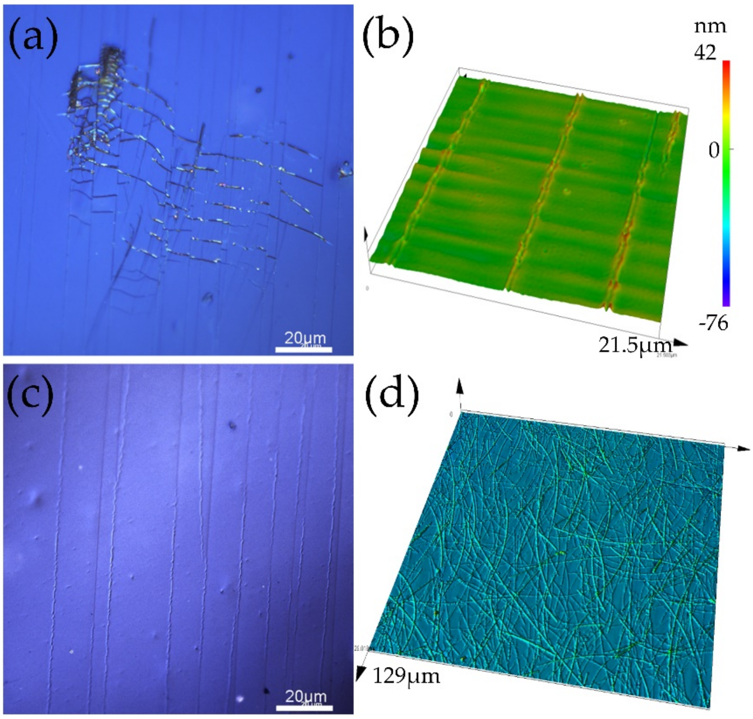

Figure S3. CLSM image of films after bending test: (**a**) reticulated cracks; (**b**) 3D scanning; (**c**) transverse cracks of the ITO-PET film after 2000 bending cycles; (**d**) 3D scanning image of AgNWs-Al_2O_3 composite film after 250,000 bending cycles.

Table S1. Changes in the optical and electrical properties of the films before and after ultrasonic treatment for 5 min.

	Sample	Pristine	After electrodeposition
	**Ultrasonic time/min**	**5 min**	**5 min**
Before	Transmittance/%	88.10	88.19
	Sheet Resistance/$\Omega\cdot sq^{-1}$	16.3	17.0

	FoM	17.28	16.74
After	Transmittance/%	98.12	92.40
	Sheet Resistance/Ω·sq^{-1}	—	160.5
	FoM	—	2.83
	FoM Change Rate/%	—	83.12
	(R-R_0)/R_0	—	8.44
	Remaining rate/%	15.80	64.35

Table S2. Relationship between sheet resistance change rate and bending cycles.

**bending cycles** / **(R_s–R_0)/R_0**	**10**	**100**	**1,000**	**10,000**	**30,000**	**70,000**	**170,000**	**250,000**
**Pristine AgNW**	−0.17	−0.26	−0.12	0.30	1.56	0.67	0.44	1.38
**Before annealing**	−0.06	0.16	−0.08	0.28	0.58	1.34	0.91	1.17
**After annealing**	0.07	−0.16	−0.21	−0.05	0.03	0.02	0.05	0.47
**ITO**	0.12	0.85	2.09	—	—	—	—	—
